# Eleutheroside E Ameliorates D-Gal-Induced Senescence in Human Skin Fibroblasts Through PI3K/AKT Signaling

**DOI:** 10.3390/cimb47110895

**Published:** 2025-10-28

**Authors:** Xiangyu Ma, Liu Han, Mengran Xu, Yuling Feng, Changsheng Liu, Yida Zhao, Min Zhang, Guanghua Xu, Xin Sun

**Affiliations:** 1 College of Pharmacy, Yanbian University, Yanji 133002, China; 2 College of Pharmacy, Jilin Medical University, Jilin 132013, Chinazhaoyida2215@163.com (Y.Z.); 3 School of Nursing, Beihua University, Jilin 132013, China

**Keywords:** Eleutheroside E, cellular senescence, β-galactosidase, PI3K/Akt pathway, human skin fibroblasts

## Abstract

Eleutheroside E (EE), a natural compound, shows promise in mitigating cellular senescence—a key factor in skin aging—though its mechanisms remain incompletely understood. This study integrated network pharmacology, molecular docking, and cellular experiments to explore the protective effects and mechanistic basis of EE against D-galactose (D-gal)-induced senescence in human skin fibroblasts (HSFs). Network pharmacology analyses suggested EE’s involvement in inflammation-related pathways, especially phosphatidylinositol 3-kinase and protein kinase B (PI3K-AKT) and hypoxia-inducible factor 1 (HIF-1) signaling, which were corroborated by molecular docking revealing strong binding affinities between EE and key targets such as hypoxia-inducible factor 1-alpha (HIF1A), AKT serine/threonine kinase 1 (AKT1), phosphatidylinositol-4,5-bisphosphate 3-kinase catalytic subunit gamma (PI3Kγ), and interleukin-6 (IL-6). Cellular assays showed that EE markedly lowered oxidative stress markers, including reactive oxygen species (ROS) and malondialdehyde (MDA), reduced senescence-associated beta-galactosidase (SA-β-gal) activity, and boosted antioxidant enzymes such as superoxide dismutase (SOD) and catalase (CAT). Additionally, EE dose-dependently inhibited apoptosis and downregulated PI3K/AKT phosphorylation as well as the B-cell lymphoma 2-associated X protein/B-cell lymphoma-2 (Bax/Bcl-2) ratio. These findings suggest that EE alleviates cellular senescence in HSFs mainly via the PI3K/AKT pathway by attenuating oxidative stress and apoptosis, highlighting its potential as a therapeutic agent for anti-aging strategies.

## 1. Introduction

Aging is an inevitable process that occurs with increasing age [1]. Compared with the aging of other less visible organs in the human body, skin aging is one of the most conspicuous signs of human aging [2], and the degree of skin aging directly reflects the overall aging status of the body [3]. As people age, the skin is constantly subjected to both internal and external stimuli, leading to progressive changes [4], including dryness, reduced dermal thickness, uneven pigmentation, decreased elasticity, and increased wrinkles [5,6,7,8,9]. Although the process of skin aging cannot be completely avoided, it can be delayed and mitigated to some extent. With the growing emphasis on life quality and skin condition, more and more research and practice are focusing on exploring natural active ingredients with anti-aging potential to slow down the process of skin aging and enhance skin health and vitality.

D-gal serves as a classical inducer of aging models [10]. Under normal physiological conditions, D-gal can combine with glucose to form lactose, which is effectively digested and absorbed by the body. However, excessive accumulation of D-gal disrupts the metabolic balance, leading to metabolic disorders that accelerate aging processes. The underlying mechanism of D-gal-induced aging is primarily associated with oxidative stress [11]. As a reducing sugar, accumulated D-gal reacts with free amino groups in proteins and peptides to form unstable Schiff bases, which subsequently undergo oxidation into stable advanced glycation end products [12]. While chronic D-gal administration in vivo disrupts systemic homeostasis, its cellular effects mainly involve the induction of intracellular redox imbalance and carbonyl stress through these glycation pathways. In skin fibroblasts, D-gal-induced oxidative damage impairs cellular function and contributes to the degradation of structural proteins like collagen and elastin, ultimately promoting skin aging [13,14]. Therefore, the D-gal-induced cellular senescence model provides a relevant platform for investigating potential anti-aging interventions [15].

Acanthopanax senticosus (commonly known as Siberian ginseng) is a widely used plant in traditional Chinese medicine, known for its dual role as both a medicinal herb and a food source [16]. EE is a natural constituent of the dried rhizomes of Acanthopanax senticosus, accounting for approximately 0.08–0.12% of the dry weight [17,18], and is one of its major active components, possessing anti-inflammatory [19], anti-fatigue [20], neuroprotective [21,22], and immunomodulatory activities [23,24]. EE has been shown to alleviate physical fatigue, enhance endurance, and exert anti-inflammatory effects by inhibiting the expression of inflammatory factors [25]. Although previous studies have demonstrated the diverse biological activities of EE, its potential protective effects against D-gal-induced senescence in HSF remain unclear. Therefore, this study aims to investigate the protective effects of EE on HSF cells by establishing an in vitro senescence model induced by D-gal, providing scientific evidence and theoretical support for its potential application in preventing and alleviating skin aging.

## 2. Materials and Methods

### 2.1. Materials

HSF cells were purchased from Qingqi Biotechnology Development Co., Ltd. (Shanghai, China). EE (HPLC ≥ 98%, CAS No. 39432-56-9, Batch No. C0069220901) was obtained from Zhongke Zhijian Biotechnology Co., Ltd. (Beijing, China). D-(+)-galactose, polyvinylidene fluoride (PVDF) membranes, and Dulbecco’s Modified Eagle Medium (DMEM) were purchased from Wuhan Boster Biological Technology Co., Ltd. (Wuhan, China). Fetal bovine serum (FBS) was purchased from Inner Mongolia Opus Biotechnology Co., Ltd. (Hohhot, China). Penicillin–streptomycin (P/S, 100×) solution was purchased from Suzhou New Cellmate Biotechnology Co., Ltd. (Suzhou, China). Cell Counting Kit-8 (CCK-8), ROS assay kit, SA-β-gal staining kit, total SOD activity assay kit (WST-8 method), MDA assay kit, CAT activity assay kit, Annexin V-FITC/PI apoptosis detection kit, enhanced chemiluminescence (ECL) kit, SDS-PAGE gel preparation kit, dual-color SDS-PAGE protein loading buffer (5×), and bicinchoninic acid (BCA) protein assay kit were all purchased from Beyotime Biotechnology Co., Ltd. (Shanghai, China). Skim milk powder was purchased from Wuhan Servicebio Technology Co., Ltd. (Wuhan, China). Trypsin digestion solution, horseradish peroxidase (HRP)-conjugated goat anti-rabbit IgG, HRP-conjugated goat anti-mouse IgG, RIPA lysis buffer (strong), phosphatase inhibitor cocktail (100×), Bax antibody (Batch No.: BC014175), Bcl-2 antibody (Batch No.: NM-009741), PI3K antibody (Batch No.: BC030815), and AKT antibody (Batch No.: BC000479) were all purchased from Wuhan Sanying Biotechnology Co., Ltd. (Wuhan, China). Phospho-AKT (p-AKT) antibody (Batch No.: AF0016) and phospho-PI3K (p-PI3K) antibody (Batch No.: AF3241) were purchased from Jiangsu Qinke Biological Research Center (Liyang, China).

### 2.2. Network Pharmacology Analysis

#### 2.2.1. Collection and Screening of EE Targets

The SMILES code of EE was sourced from PubChem. Potential targets were identified using three databases—SEA, SwissTargetPrediction, and Super-PRED. To ensure the relevance of our findings to human biology, only targets explicitly annotated as “Homo sapiens” or derived from human-specific data were considered and retrieved from all three databases. To balance sensitivity and confidence, we employed database-specific selection criteria: all targets from SEA were included for broad coverage; SwissTargetPrediction targets with probability scores > 0 were retained for maximum sensitivity; and only Super-PRED targets exceeding the 60% probability threshold were included to ensure high-confidence predictions. The final target set was generated by merging and deduplicating all qualified targets from these sources.

#### 2.2.2. Collection and Screening of Targets Related to D-Gal-Induced Senescence

Potential targets associated with D-gal-induced senescence were identified by searching the GeneCards (https://www.genecards.org/ (accessed on 5 April 2025)) and OMIM (https://omim.org/ (accessed on 5 April 2025)) databases using the keyword “D-gal-induced senescence.” These databases provide comprehensive information on genes and diseases, facilitating the identification of targets involved in the process of D-gal-induced senescence.

#### 2.2.3. Target Prediction for Drug–Disease Associations

The targets of EE components and those induced by D-galactose-induced senescence were imported into the bioinformatics online platform to generate Venn diagrams and perform visualization analysis. Subsequently, these targets were imported into Cytoscape version 3.8.2 to construct a “drug–disease–target” network visualization. This visualization reflects the complex relationships among these three entities.

#### 2.2.4. Protein–Protein Interaction (PPI) Network Construction and Hub Target Screening

The intersecting targets between EE components and D-gal-induced senescence were imported into the STRING database (https://string-db.org/ (accessed on 7 April 2025)) to construct a PPI network, followed by topology analysis and visualization using Cytoscape 3.8.2 software, where the top 10 targets ranked by degree value were identified as core hub targets.

#### 2.2.5. Gene Ontology (GO) Enrichment and Kyoto Encyclopedia of Genes and Genomes (KEGG) Pathway Analysis

To gain a deeper understanding of the molecular mechanisms underlying drug–disease interactions, we uploaded the intersecting genes of drug–disease to the DAVID database (https://davidbioinformatics.nih.gov (accessed on 13 April 2025)) for GO analysis. GO annotations are primarily categorized into three main classes: Biological Process (BP), Cellular Component (CC), and Molecular Function (MF). Additionally, KEGG pathway analysis was conducted to identify the comprehensive functions and biological relevance of the candidate targets, thereby predicting the mechanisms of EE in disease treatment.

#### 2.2.6. Molecular Docking

The three-dimensional (3D) protein structure of EE was downloaded from the Research Collaboratory for Structural Bioinformatics Protein Data Bank (RCSB PDB, https://www.rcsb.org/ (accessed on 3 May 2025)). The modified ligands were removed and water molecules were eliminated using the PyMOL (version 2.5.0). The 3D structure of the core component was downloaded from the PubChem database and optimized using Chem3D (version 21.0). Molecular docking of the active component with the core target was performed using AutoDock (version 4.2.6), and the docking mode between the core target and the active component was visualized using PyMOL (version 2.5.0).

### 2.3. Experimental

#### 2.3.1. Cell Culture and Treatment

HSF cells were cultured in DMEM complete medium supplemented with 10% FBS and 1% P/S at 37 °C in a humidified 5% CO_2_ incubator. Upon reaching 80–90% confluence, cells were washed 1–2 times with PBS, harvested using 0.25% trypsin-EDTA solution, and subcultured at a 1:3 split ratio.

#### 2.3.2. Cell Grouping and Pharmacological Intervention

Cells were divided into four groups: control group, D-gal model group (120 mM D-gal), D-gal + low-dose EE group (50 μmol/L EE), and D-gal + high-dose EE group (200 μmol/L EE). The D-gal working solution (120 mM) was prepared by dissolving 216.3 mg of D-gal in 10 mL of complete cell culture medium, followed by filter sterilization through a 0.22 μm membrane. For assays conducted in 96-well plates (CCK-8 assay), cells were seeded at 3 × 10^3^ cells/well and cultured for 24 h until full adherence. After medium removal, EE-treated groups received 200 μL complete medium containing respective EE concentrations (50/200 μmol/L), while control and model groups received an equal volume of complete medium for 24 h. Following medium replacement, all groups except the control were treated with 120 mM D-gal medium for 12 h. For experiments performed in 6-well plates, including measurements of SOD, CAT, MDA, and ROS levels, β-galactosidase staining, flow cytometry, and protein extraction, HSF cells were plated at 4.5 × 10^5^ cells/well (2 mL/well), cultured for 12 h, washed twice with PBS, and treated as above.

#### 2.3.3. CCK-8 Assay

The cytotoxicity of EE and the modeling concentration of D-galactose (D-gal) were determined using the Cell Counting Kit-8 (CCK-8) assay. Briefly, HSF cells were seeded in 96-well plates at a density of 3 × 10^3^ cells per well and allowed to adhere overnight. For EE cytotoxicity assessment, cells were treated with a range of EE concentrations (0, 50, 100, 200, 300, 400, and 500 μmol/L) for 24 h. For the D-gal-induced aging model establishment, cells were treated with various concentrations of D-gal (0, 100, 120, 140, 160, 180, and 200 mM) for 12 h. After the respective treatments, the culture medium was replaced with 100 μL of fresh medium containing 10% CCK-8 reagent, and the cells were incubated at 37 °C for 2 h. The absorbance was measured at 450 nm using a microplate reader. Cell survival rate = [(OD of experimental wells − OD of blank wells)/(OD of control wells − OD of blank wells)] × 100%. The optimal concentration for subsequent experiments was selected based on the viability results.

#### 2.3.4. Measurement of SOD Activity, CAT Activity, and MDA Content in HSF Using Standardized Spectrophotometric Assays

Following interventions, HSF cells were collected and processed according to manufacturer’s protocols to quantify MDA levels, SOD activity, and CAT activity. Each independent experiment was performed with quintuplicate technical replicates per group. The data presented are pooled from three independent biological replicates (*n* = 3).

#### 2.3.5. Quantification of SA-β-Gal Positive Area in HSF Using Standardized Chromogenic Staining Protocol

Following drug interventions, SA-β-gal activity was measured by washing cells 1–2 times with PBS, fixing with 1 mL SA-β-gal staining fixative for 15 min, incubating with 1 mL staining working solution overnight at 37 °C, and, finally, visualizing and photographing stained cells under an inverted fluorescence microscope. The percentage of SA-β-gal positive area was quantified, and the data shown are representative of three independent biological replicates (*n* = 3).

#### 2.3.6. Quantification of Intracellular ROS Levels in HSF Using 2′,7′-Dichlorodihydrofluorescein Diacetate (DCFH-DA) Fluorescent Probe

After the drug interventions, the culture medium was aspirated and the cells were washed twice with phosphate-buffered saline (PBS). Subsequently, the cells were incubated with 10 μmol/L 2′,7′-dichlorodihydrofluorescein diacetate (DCFH-DA) probe (diluted 1:1000 in serum-free medium) at 37 °C for 20 min in the dark. Following incubation, the cells were thoroughly rinsed three times with serum-free medium to remove any residual probe. The intracellular ROS levels were assessed using fluorescence microscopy. Fluorescence intensity was quantified using ImageJ software (version 1.54p, National Institutes of Health, Bethesda, MD, USA) and expressed as relative fluorescence units (RFUs) normalized to the control group. Each experiment was performed in triplicate and repeated independently at least three times to ensure reproducibility.

#### 2.3.7. Flow Cytometry Analysis of Apoptosis in HSF Cells

Following drug interventions, the culture medium was removed and transferred to centrifuge tubes. Cells were washed twice with PBS, digested with 500 μL trypsin, and the enzymatic digestion was neutralized by adding back the previously collected conditioned medium. After gentle pipetting to detach cells, the suspension was centrifuged at 1000 rpm for 5 min. The pellet was resuspended in PBS for cell counting prior to subsequent kit-specific procedures, followed by flow cytometric analysis.

#### 2.3.8. Western Blot Analysis of Senescence-Associated Protein Expression in HSF Cells

Following interventions, cells were lysed with RIPA buffer containing phosphatase inhibitor cocktail (100×). Protein concentrations were determined by BCA assay, mixed with 4× loading buffer at 1:4 ratio, and denatured at 100 °C for 10 min. Proteins were electrophoresed at 100 V, transferred to PVDF membranes at 240 mA, and blocked with 5% skim milk for 2 h. After TBST washes, membranes were incubated overnight at 4 °C with primary antibodies against PI3K (1:5000), p-PI3K (1:1000), AKT (1:3000), p-AKT (1:1000), Bax (1:5000), Bcl-2 (1:3000), and β-actin (1:5000). Following secondary antibody incubation (species-matched, 2 h at room temperature) and TBST washes, protein bands were visualized and quantified using ImageJ software.

### 2.4. Statistical Analysis

Data are expressed as mean ± standard deviation (SD) from three independent experiments (*n* = 3). All statistical analyses were performed using GraphPad Prism software (version 9.5). The assumptions of normality and homogeneity of variance were checked for all datasets. Comparisons among multiple groups were analyzed by one-way analysis of variance (ANOVA). When a significant overall effect was found (*p* < 0.05), Dunnett’s post hoc test was applied specifically for comparisons against the control group. *p* < 0.05 was considered statistically significant.

### 2.5. Writing Assistance Disclosure

During the process of writing the manuscript, we used the DeepSeek AI assistant (Deepseeking-V2) to optimize the language expression of the manuscript. We input some sentences of the manuscript into the AI system for grammar correction and sentence structure optimization. For example, “Please, without changing the meaning of the sentence, Optimize the grammar and clarity of the following sentence in a scientific writing style: [Sentence].” All the scientific content in this article has been independently conceived, completed and verified by us. AI is only used to enhance the readability of the text, and the final content has been reviewed by all authors, who are fully responsible for its scientific nature.

## 3. Results

### 3.1. Target Collection and Prediction

**Figure 1:**
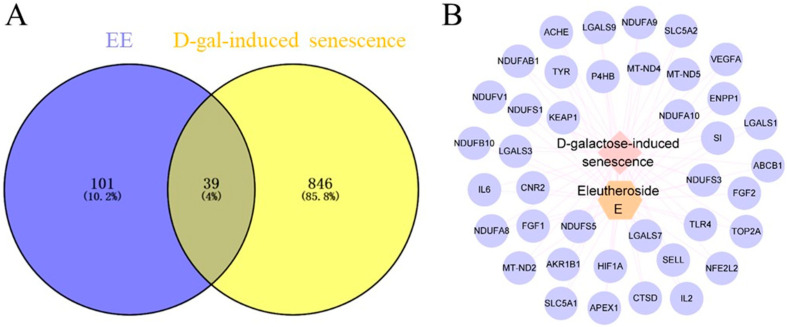
Target collection and prediction results. (**A**) Venn diagram of overlapping targets between EE and D-gal-induced senescence. (**B**) Drug–disease–target network visualization.

A total of 140 potential targets of EE were identified through database screening (SEA, SUPER-PRED, and SWISS) followed by deduplication. Additionally, 885 targets associated with D-gal-induced senescence were retrieved from the GeneCards and OMIM databases. Intersection analysis between EE-related targets and disease-associated genes revealed 39 overlapping targets (Figure 1A), which were considered potential therapeutic targets for EE in mitigating D-gal-induced senescence. To elucidate the key components of EE in D-gal-induced senescence treatment, drug–disease–target data were compiled into “network.xlsx” and “tape.xlsx” files. These files were imported into Cytoscape 3.8.2 to construct a drug–disease–target network (Figure 1B). In the network, targets are represented by circles, drugs by hexagons, and diseases by diamonds.

### 3.2. Construction of the PPI Network

**Figure 2:**
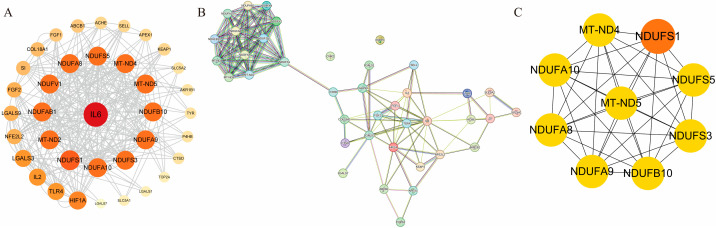
PPI network diagram. (**A**) Target screening. (**B**) PPI network visualization. (**C**) Hub gene identification.

After intersecting the target genes of EE with disease-related genes, 39 common target genes were identified as potential interactive mediators of EE’s therapeutic effects (Figure 2A). These overlapping genes were subsequently imported into the STRING database (https://string-db.org/ (accessed on 7 April 2025)) for PPI prediction, and the resulting interaction network was visualized using Cytoscape 3.8.2 (Figure 2B). The cytoHubba plugin was employed to identify the top 10 hub targets based on degree centrality (Figure 2C). The highest-ranking core targets included NDUFS1 (NADH: Ubiquinone oxidoreductase core subunit S1), MT-ND4 (Mitochondrially Encoded NADH:Ubiquinone Oxidoreductase Core Subunit 4), MT-ND5 (Mitochondrially Encoded NADH:Ubiquinone Oxidoreductase Core Subunit 5), NDUFS5 (NADH:ubiquinone oxidoreductase subunit S5), and NDUFS3 (NADH:ubiquinone oxidoreductase core subunit S3), among others.

### 3.3. GO and KEGG Pathway Enrichment Analysis

**Figure 3:**
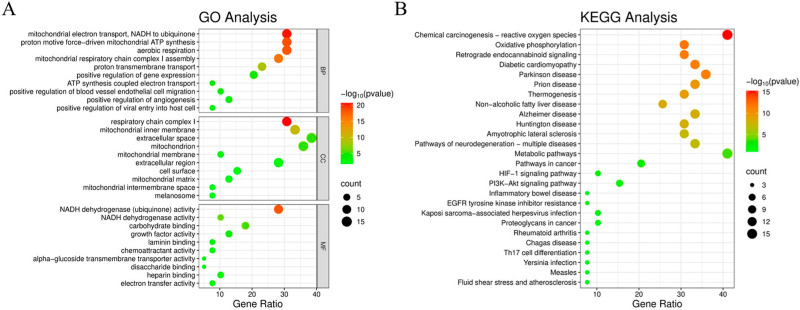
Bubble plots of GO and KEGG enrichment analyses. (**A**) GO analysis. (**B**) KEGG analysis.

GO functional enrichment analysis of drug–disease intersecting genes was performed using the DAVID database, with the top 10 ranked terms in biological processes (BPs), cellular components (CCs), and molecular functions (MFs) visualized in bubble plots (Figure 3A). The BP terms showed significant associations with mitochondrial electron transport (NADH to ubiquinone), proton motive force-driven mitochondrial ATP synthesis, aerobic respiration, mitochondrial respiratory chain complex I assembly, proton transmembrane transport, positive regulation of gene expression, positive regulation of angiogenesis, ATP synthesis coupled electron transport, positive regulation of blood vessel endothelial cell migration, and positive regulation of viral entry into host cell. The CC analysis revealed close relationships with respiratory chain complex I, mitochondrial inner membrane, extracellular space, mitochondrion, mitochondrial membrane, extracellular region, cell surface, mitochondrial matrix, mitochondrial intermembrane space, and melanosome. For MF, the most relevant terms included NADH dehydrogenase (ubiquinone) activity, NADH dehydrogenase activity, carbohydrate binding, alpha-glucoside transmembrane transporter activity, growth factor activity, laminin binding, chemoattractant activity, electron transfer activity, heparin binding, and disaccharide binding.

KEGG pathway enrichment analysis was performed using the DAVID database, with the top 30 pathways visualized in a bubble plot (Figure 3B) where darker bubble color indicates higher pathway significance (−log10[*p*-value]) and larger bubble diameter represents greater numbers of enriched genes. The analysis revealed that EE’s anti-aging targets against D-gal-induced senescence were primarily enriched in chemical carcinogenesis–reactive oxygen species, metabolic pathways, and several inflammation-related signaling pathways.

### 3.4. Molecular Docking Results

**Figure 4:**
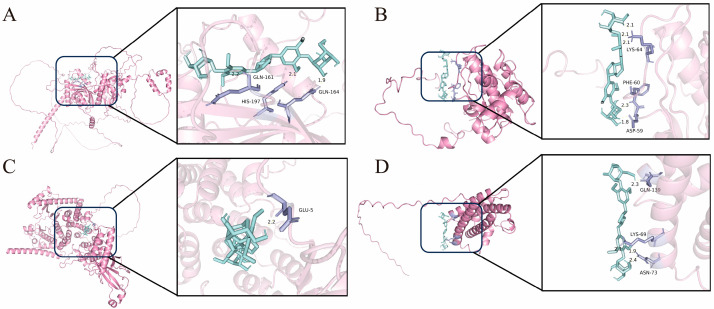
Molecular docking results. (**A**) HIF1A-EE. (**B**) AKT1-EE. (**C**) PI3Kγ-EE. (**D**) IL-6-EE.

Molecular docking studies were performed to evaluate the binding interactions between EE and core targets identified through network pharmacology analysis. Based on the core targets and pathways identified, four protein structures were selected from the Protein Data Bank (PDB): HIF1A (PDB: 2CGO), AKT1 (PDB: 2UZT), PI3Kγ (PDB: 5G2N), and IL-6 (PDB: 7PHS). This selection specifically aimed to provide structural insights for subsequent experimental validation, particularly for investigating the PI3K/AKT signaling pathway. The corresponding binding energies were calculated (Table 1), where a lower binding energy indicates a stronger interaction between the compound and the target protein, suggesting enhanced efficacy and greater stability of the complex [26]. As shown in Figure 4 and Table 1, EE demonstrated the strongest predicted interaction with AKT1, with a binding energy of −5.92 kcal/mol, providing a structural basis for our subsequent investigation into its effects on PI3K/AKT phosphorylation.

### 3.5. Cytotoxicity Evaluation of EE by CCK-8 Assay and Its Intervention in Oxidative Stress-Induced Senescence Model

**Figure 5:**
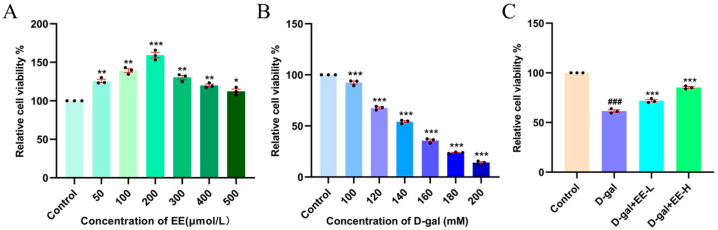
Effects of EE on HSF cell viability and protection against D-Gal-induced senescence. (**A**) Effects of different EE concentrations on HSF cell viability. Data were analyzed using one-way ANOVA followed by Dunnett’s post hoc test. * *p* < 0.05, ** *p* < 0.01, *** *p* < 0.001 vs. control group. (*n* = 3, mean ± SD). (**B**) Establishment of D-gal-induced oxidative stress senescence model in HSF cells. Data were analyzed using one-way ANOVA followed by Dunnett’s post hoc test. *** *p* < 0.001 vs. control group. (*n* = 3, mean ± SD). (**C**) Protective effects of EE pretreatment on cell viability in D-gal-induced senescence model. Data were analyzed using one-way ANOVA followed by Dunnett’s post hoc test. ^###^ *p* < 0.001 vs. control group; *** *p* < 0.001 vs. model group. (dots (·) represent *n* = 3, mean ± SD).

Cell viability, a key indicator for assessing cellular damage [27], was measured in HSF cells treated with varying concentrations of EE (50–500 μmol/L) using CCK-8 assay. As shown in Figure 5A, cell viability remained at or above 100% across all tested concentrations, indicating the absence of cytotoxicity at concentrations up to 500 μmol/L. Treatment with 200 μmol/L EE significantly enhanced metabolic activity by 43 ± 6.63% (*p* < 0.0001) compared to the control group. Although the 500 μmol/L concentration showed no cytotoxic effects, it did not demonstrate a superior enhancement of metabolic activity compared to the 200 μmol/L group. Therefore, based on the consideration that 200 μmol/L exhibited the most significant enhancement of cell viability while 50 μmol/L represented the threshold concentration for its beneficial effects, these two concentrations—50 (low) and 200 (high) μmol/L—were selected for subsequent experiments. To establish an aging model, HSF cells were treated with D-gal [28]. As shown in Figure 5B, treatment with 120 mM D-gal significantly reduced cell viability to 60–70% (*p* < 0.001). This concentration, which is close to the 20 g/L used in previous studies [29,30,31], effectively induced cellular senescence without causing marked cytotoxicity. Therefore, 120 mM D-gal was selected for all subsequent experiments. In Figure 5C, D-gal-treated model cells showed decreased viability (61.42 ± 0.27%, *p* < 0.0001), whereas pretreatment with 50 and 200 μmol/L EE dose-dependently increased viability to 71.89 ± 2.06% (*p* = 0.0002) and 85.26 ± 1.61% (*p* < 0.0001), respectively, demonstrating significant protective effects against cellular senescence.

### 3.6. Effect of EE on Oxidative Stress Markers in HSF Cells

**Figure 6:**
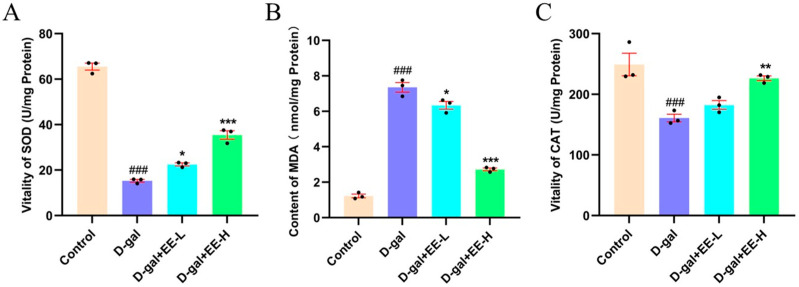
Effect of EE on antioxidant capacity of senescent HSF cells. (**A**) SOD activity. (**B**) MDA content. (**C**) CAT activity. Data were analyzed using one-way ANOVA followed by Dunnett’s post hoc test. ^###^ *p* < 0.001 vs. control group; * *p* < 0.05, ** *p* < 0.01, *** *p* < 0.001 vs. model group. (*n* = 3, mean ± SD).

SOD and CAT are crucial antioxidant enzymes in organisms that primarily function to decompose peroxides and mitigate oxidative stress [32]. Enhancing the activity of these enzymes can effectively delay aging processes. SOD, as an important antioxidant enzyme, exerts a strong antioxidant effect by catalyzing the decomposition of superoxide free radicals into more stable oxygen and hydrogen peroxide, eliminating ROS in cells [33]. MDA, a product of lipid peroxidation in cell membranes, serves as a reliable biomarker for oxidative stress [34]. CAT primarily decomposes hydrogen peroxide into water and oxygen, thereby reducing cellular damage caused by hydrogen peroxide [35]. In this study, the antioxidant capacity of EE against D-galactose-induced oxidative damage in HSF cells was evaluated by measuring SOD and CAT activities as well as MDA levels. As shown in Figure 6A, compared to the control group, the model group exhibited significantly decreased SOD activity (15.31 ± 1.02%, *p* < 0.0001), indicating oxidative stress-mediated suppression. Treatment with 50 μmol/L EE increased SOD activity to 22.51 ± 1.09% (*p* =0.0021 vs. model), while 200 μmol/L EE showed more pronounced elevation to 35.42 ± 3.18% (*p* < 0.0001 vs. model). Figure 6B demonstrates that the model group showed significantly increased MDA content (7.35 ± 0.47, *p* < 0.0001) compared to the control group. EE treatment at 50 μmol/L reduced MDA levels to 6.34 ± 0.58 (*p* = 0.0116), while 200 μmol/L EE further decreased to 2.72 ± 0.12 (*p* < 0.0001). Additionally, as illustrated in Figure 6C, CAT activity in the model group decreased by 40.23 ± 3.15% (*p* = 0.0009) compared to the control group. Treatment with 50 μmol/L EE increased CAT activity by 15.87 ± 2.25% (*p* = 0.0060), while 200 μmol/L EE showed a more pronounced 42.39 ± 5.13% increase (*p* < 0.0001). These findings suggest that EE exerts anti-aging effects by upregulating SOD and CAT expression while reducing MDA accumulation, thereby protecting HSF cells against D-gal-induced oxidative damage.

### 3.7. Measurement of Cellular Antioxidant Enzyme Activities

**Figure 7:**
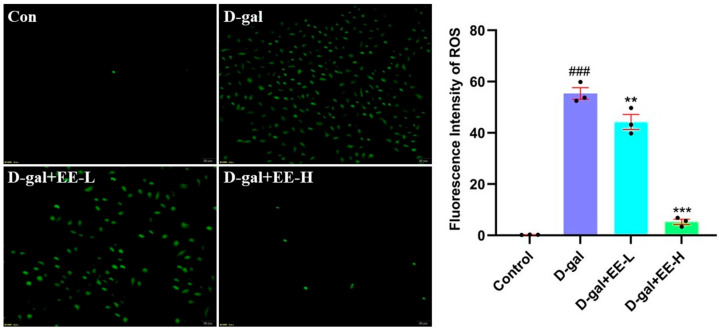
Antioxidant effects of EE against D-gal-induced oxidative stress. The figure shows representative images (**left**) of intracellular ROS detected by DCFH-DA staining and the quantitative analysis (**right**) of fluorescence intensity. Data were analyzed using one-way ANOVA followed by Dunnett’s post hoc test. ^###^ *p* < 0.001 vs. control group; ** *p* < 0.01, *** *p* < 0.001 vs. model group. Scale: 20 µm. (*n* = 3, mean ± SD).

Excessive ROS generation may directly cause cellular damage by oxidizing cellular proteins, lipids, and DNA, or indirectly impair cellular functions through disruption of normal signaling pathways and gene regulation [36,37]. As shown in Figure 7, D-gal-treated model cells exhibited a significant increase in ROS levels (55.36 ± 3.81 RFU, *p* < 0.0001). In contrast, pretreatment with EE resulted in a dose-dependent attenuation of the fluorescence signal, reducing it to 44.23 ± 4.96 RFU (50 μmol/L, *p* = 0.0087) and 5.27 ± 1.72 RFU (200 μmol/L, *p* < 0.0001). While the pronounced reduction at 200 μmol/L suggests potent bioactivity, the possibility of minor signal interference, such as fluorescence quenching by EE itself, cannot be entirely excluded. Nevertheless, the consistent dose–response relationship observed across three independent experiments supports that EE effectively mitigates D-gal-induced ROS overproduction.

### 3.8. Effect of EE on D-Gal-Induced Cellular Senescence

**Figure 8:**
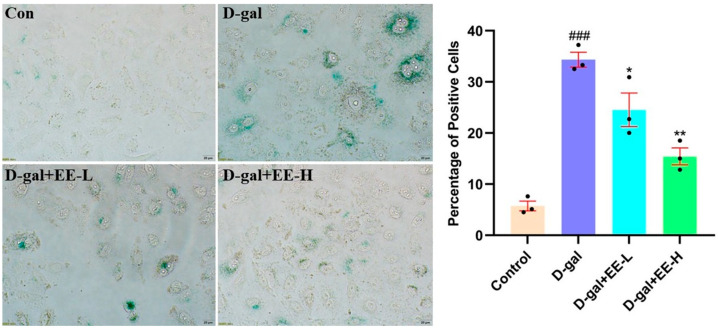
Quantification of SA-β-gal positive cells. The figure shows representative images (**left**) of SA-β-gal staining and the quantification (**right**) of SA-β-gal positive cells. Data were analyzed using one-way ANOVA followed by Dunnett’s post hoc test. ^###^ *p* < 0.001 vs. control group; * *p* < 0.05, ** *p* < 0.01 vs. model group. Scale: 20 µm. (*n* = 3, mean ± SD).

As shown in Figure 8, the model group exhibited a significant increase in SA-β-Gal-positive cells (34.3 ± 2.4%, *p* < 0.0001 vs. control) with enhanced blue staining. EE pretreatment demonstrated dose-dependent anti-senescence effects, reducing SA-β-Gal positivity to 24.5 ± 5.5% at 50 μmol/L (*p* = 0.0230) and further to 15.4 ± 3.0% at 200 μmol/L (*p* = 0.0005), along with decreased staining intensity. Increased SA-β-galactosidase activity serves as a crucial biomarker of cellular senescence and represents the gold standard for senescence detection [38]. These results indicate that EE effectively attenuates D-gal-induced HSF cell senescence, further confirming its anti-aging potential.

### 3.9. Effect of EE on D-Gal-Induced Apoptosis

**Figure 9:**
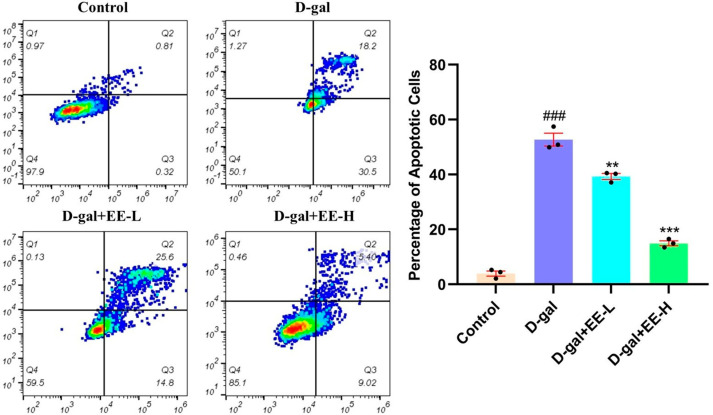
Quantification of apoptotic cells by flow cytometry with Annexin V-FITC/PI staining. The figure shows representative scatter plots (**left**) of flow cytometry analysis with Annexin V-FITC/PI staining and the quantification (**right**) of apoptotic cells. Data were analyzed using one-way ANOVA followed by Dunnett’s post hoc test. ^###^ *p* < 0.001 vs. control group; ** *p* < 0.01, *** *p* < 0.001 vs. model group. (*n* = 3, mean ± SD).

As shown in Figure 9, D-gal treatment significantly induced cellular apoptosis (52.7 ± 4.0%, *p* < 0.0001 vs. control), confirming the crucial role of apoptosis in senescence progression. EE pretreatment at 50 μmol/L reduced apoptosis rates to 39.3 ± 1.8% (*p* = 0.0005 vs. model), while 200 μmol/L EE further decreased apoptosis to 14.9 ± 1.6% (*p* < 0.0001 vs. model), demonstrating dose-dependent cytoprotection with 25.4% and 71.7% reductions relative to model levels, respectively. These results suggest EE exerts anti-aging effects potentially through modulation of apoptotic pathways to inhibit HSF cell death.

### 3.10. Western Blot Experiments Were Conducted to Investigate the Effects of EE on the Expression of PI3K/Akt Pathway-Related Proteins in HSF Cells

**Figure 10:**
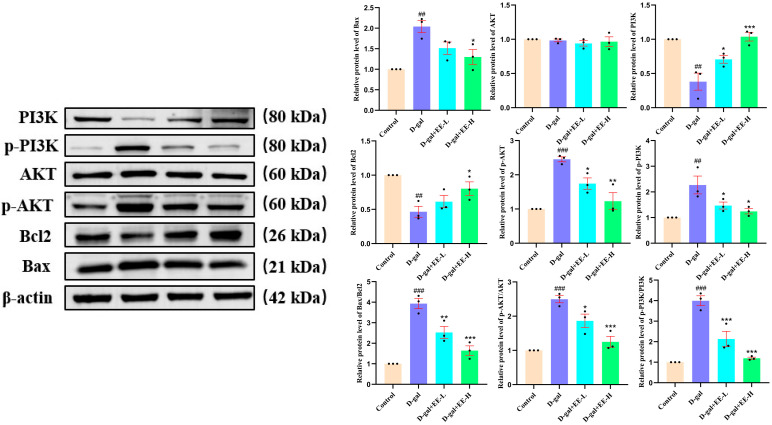
Effect of EE on the PI3K/AKT signaling pathway in D-gal-induced HSF cell senescence. The figure shows representative Western blot bands (**left**) and the corresponding quantitative analysis (**right**) of key proteins in the PI3K/AKT pathway. β-actin was used as the loading control. Data were analyzed using one-way ANOVA followed by Dunnett’s post hoc test. ^##^ *p* < 0.01, ^###^ *p* < 0.001 vs. control group; * *p* < 0.05, ** *p* < 0.01, *** *p* < 0.001 vs. model group. (*n* = 3, mean ± SD).

Western blot analysis demonstrated significant dysregulation of apoptotic markers in D-gal-induced senescence, showing markedly increased Bax expression (*p* = 0.0019) and decreased Bcl-2 levels (*p* = 0.0032) compared to controls. Concurrent analysis revealed substantial activation of PI3K/AKT signaling pathway, evidenced by elevated p-PI3K/PI3K (*p* = 0.0002) and p-AKT/AKT ratios (*p* < 0.0001). EE treatment effectively counteracted these molecular alterations, significantly reducing Bax expression (*p* = 0.0139), restoring Bcl-2 levels (*p* = 0.0379), and normalizing both p-PI3K/PI3K (*p* < 0.0001) and p-AKT/AKT ratios (*p* = 0.0006). These findings collectively indicate EE’s capacity to counteract D-gal-induced dysregulation of apoptotic proteins and attenuate PI3K/AKT hyperactivation (Figure 10). This mechanistic insight corroborates flow cytometry data showing reduced apoptosis rates, substantiating EE’s multi-target anti-apoptotic and anti-senescence effects.

## 4. Discussion

This study systematically investigated the protective mechanisms of EE against D-gal-induced senescence in HSF through an integrated approach combining network pharmacology and experimental validation. Our principal findings demonstrate that EE exerts multi-faceted anti-senescence effects primarily through three interconnected mechanisms: (1) ameliorating oxidative stress by reducing ROS and MDA levels while restoring SOD and CAT activities; (2) suppressing apoptosis via modulating the Bax/Bcl-2 ratio; and (3) most significantly, attenuating D-gal-induced hyperactivation of the PI3K-AKT signaling pathway. Network pharmacology predictions and molecular docking further identified key targets (including HIF1A, AKT1, PI3Kγ, and IL-6) and implicated inflammation-related pathways in EE’s action. Collectively, these results provide a scientific foundation for developing EE as a novel senotherapeutic intervention.

EE, a triterpenoid saponin present in the roots and rhizomes of Acanthopanax senticosus, exhibits diverse pharmacological properties including antioxidant, anti-inflammatory, and anti-apoptotic activities [39]. Recent advances in phytochemical research have highlighted its emerging potential as an anti-aging therapeutic agent. D-gal, a well-established senescence inducer, mimics endogenous oxidative stress and aging processes to trigger cellular senescence [40]. During its metabolic processing, D-gal generates excessive ROS, exacerbating intracellular oxidative stress and subsequently activating both apoptotic pathways and senescence-associated signaling cascades [41]. Experimental data demonstrated that D-gal-treated HSF exhibited hallmark senescent phenotypes, including elevated intracellular ROS levels, increased MDA content, reduced SOD and CAT activities, along with enhanced senescence-associated SA-β-gal positivity. Notably, EE administration significantly reversed these senescence-associated biomarkers, substantiating its potent anti-senescence efficacy.

Apoptosis, a genetically programmed cell death process, serves as a critical mechanism for maintaining cellular and tissue homeostasis, regulating growth and differentiation, and controlling tissue repair [42]. The intrinsic apoptotic pathway is predominantly governed by the dynamic equilibrium between pro-apoptotic Bax and anti-apoptotic Bcl-2 members of the BCL-2 protein family, whose interplay determines cellular fate [43]. Our findings demonstrate that EE significantly attenuated D-gal-induced apoptosis by coordinately downregulating pro-apoptotic Bax while upregulating anti-apoptotic Bcl-2 expression, thereby decelerating cellular senescence progression. Flow cytometric quantification further validated EE’s dose-dependent anti-apoptotic efficacy, with high-dose treatment achieving 37.77% reduction in apoptotic rate (*p* < 0.001).

The PI3K-AKT signaling pathway plays a pivotal role in regulating cellular survival, proliferation, apoptosis, and senescence [44]. Dysregulated activation of this pathway is closely associated with various pathological conditions, including cancer, neurodegenerative disorders, and aging [45]. During cellular senescence, hyperactivation of PI3K-AKT signaling promotes apoptotic progression and exacerbates oxidative stress, thereby accelerating senescence [46]. In our D-gal-induced HSF model, significantly elevated phosphorylation ratios of p-PI3K/PI3K and p-AKT/AKT confirmed pathway activation. Notably, EE treatment effectively attenuated these phosphorylated protein levels, attenuating PI3K-AKT hyperactivation and consequently exhibiting anti-senescence properties.

Network pharmacology represents a systematic approach for elucidating drug mechanisms by constructing drug–target–disease interaction networks to predict potential therapeutic targets and pathways [47]. In this study, network pharmacological analysis predicted anti-senescence targets of EE, identifying intersectional targets between EE and D-gal-induced senescence models. PPI network construction and subsequent hub target screening revealed core targets underlying EE’s anti-aging effects. KEGG pathway enrichment analysis demonstrated significant association of these targets with inflammation-related pathways, such as the PI3K-AKT and HIF-1 signaling, suggesting that EE may exert anti-senescence effects through modulation of inflammatory cascades. Molecular docking validated strong binding affinities between EE and key targets (HIF1A, AKT1, PI3Kγ, and IL-6), indicating these molecules as critical mediators. These findings not only provide mechanistic insights into EE’s anti-aging properties but also propose novel targets for future therapeutic development.

While this study provides insights into the anti-senescence mechanism of EE, certain limitations should be considered. Our findings are primarily based on a D-gal-induced cellular model, which may not fully recapitulate the complexity of natural aging in vivo. Future research directions should include validating these findings in animal models of physiological aging and exploring the involvement of other predicted pathways, such as HIF-1 signaling, through multi-omics approaches. Regarding potential clinical applications, the demonstrated efficacy of EE in mitigating oxidative stress, apoptosis, and PI3K-AKT hyperactivation positions it as a promising candidate for developing novel senotherapeutic interventions. Future efforts should focus on optimizing its bioavailability, assessing its long-term safety in preclinical models, and exploring its potential in age-related dermatological conditions or systemic age-related disorders.

## 5. Conclusions

**Figure 11:**
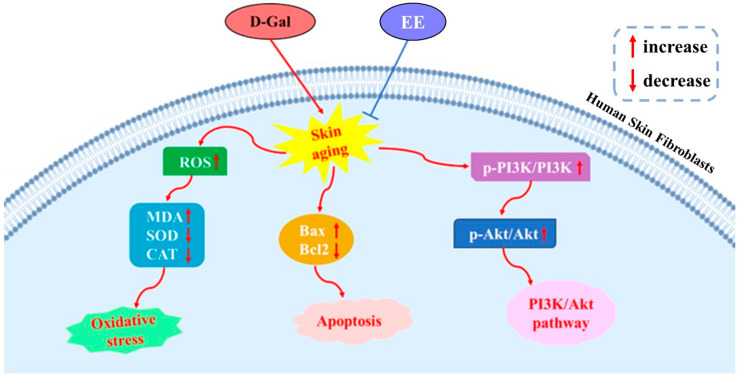
EE attenuates D-gal-induced cellular senescence in HSF by modulating oxidative stress, apoptosis, and the PI3K-AKT pathway.

This study comprehensively elucidates that EE effectively counteracts D-gal-induced senescence in human skin fibroblasts through multi-modal mechanisms. By integrating network pharmacology prediction with experimental validation, we demonstrate that EE mitigates oxidative damage (via ROS scavenging and SOD/CAT enhancement), inhibits apoptosis (via Bax/Bcl-2 modulation), and suppresses PI3K-AKT hyperactivation, thereby orchestrating anti-senescence effects (Figure 11). These findings not only advance the mechanistic understanding of EE’s anti-aging potential but also highlight its promise as a novel senotherapeutic agent targeting oxidative stress, apoptosis, and dysregulated signaling pathways.

## Figures and Tables

**Table 1:** Molecular docking binding energy data.

Ingredients	Target Point	Binding Energy (kJ/mol)
EE	HIF1A	−4.62
	AKT1	−5.92
	PI3Kγ	−5.13
	IL-6	−5.3

## Data Availability

The original contributions presented in the study are included in the article; further inquiries can be directed to the corresponding author.

## Abbreviations

The following abbreviations are used in this manuscript:

EE	Eleutheroside E
HSF	Human Skin Fibroblast
BP	Biological Process
CC	Cellular Component
MF	Molecular Function
CAT	Catalase
SOD	Superoxide dismutase
ROS	Reactive oxygen species
MDA	Malondialdehyde
PPI	protein–protein interaction
